# Self-Efficacy, Flow, Affect, Worry and Performance in Elite World Cup Ski Jumping

**DOI:** 10.3389/fpsyg.2018.01215

**Published:** 2018-07-17

**Authors:** Vegard H. Sklett, Håvard W. Lorås, Hermundur Sigmundsson

**Affiliations:** ^1^Department of Neuromedicine and Movement Science, Faculty of Medicine and Health Sciences, Norwegian University of Science and Technology, Trondheim, Norway; ^2^Department of Psychology, Faculty of Social and Educational Sciences, Norwegian University of Science and Technology, Trondheim, Norway; ^3^Department of Sport Science and Physical Education, Reykjavik University, Reykjavik, Iceland

**Keywords:** self-efficacy, flow, positive affect, negative affect, worry, ski jumping, performance

## Abstract

The present study investigated the relationship between self-efficacy, flow, positive- and negative affect, worry and ski jumping performance, as well as the degree of influence these psychological factors have on ski jumping performance in specific competitions and overall World Cup ranking. World Cup ski jumpers (*N* = 40) responded to four questionnaires in the middle of the World Cup season, reporting their subjective experience during a competitive setting over a period of three consecutive days. Social Cognitive Theory (SCT) and Flow Theory was used as main conceptual frameworks. Self-efficacy was moderately related to ski jumping performance, both overall World Cup ranking (*r* = −0.37) and the results from the first out of three individual ski flying competitions (*r* = −0.36) and explained approximately 14% of the variance in the overall World Cup. Flow was moderately related to ski jumping performance, both overall World Cup ranking (Flow-Focus) (*r* = −0.34), and individual ski flying results from the first competition (Flow-Arousal) (*r* = −0.36). The Flow-Arousal explained approximately 13% of the variance in ski flying results. Worry was highly related to ski jumping performance in the second (*r* = 0.60) and third (*r* = 0.52) competition, indicating that approximately 36 and 27% of the variance in ski flying results could be accounted for by levels of worry, respectively. Negative affect was moderately related to ski flying performance (*r* = 0.34). These results show that psychological factors that regulate emotional states may be of importance for World Cup ski jumping performance, and that appropriate coping strategies, constructive mindset and motivation, appears to be essential in this regard. To our knowledge, this is the first study on the relationship between these psychological factors and ski jumping performance among World Cup athletes. The study adds important information about some of the dynamic features of emotional and psychological mechanisms involved during elite ski jumping performance.

## Introduction

Ski jumping is a multi-factorial sport as it requires control of technical, coordinative, and physiological (fitness, power, arousal and tension regulation) aspects. These domains and skills are in a mutually influencing relationship and form the basis of a comprehensive understanding of performance-enhancing factors for athletic performance (Martin, 1982). In particular, appropriate emotional states are also required for optimizing developmental processes, and thus, among the strongest predictors of success (Hanin, 2000; Groslambert et al., 2003; Hardy et al., 2004; Cox et al., 2010; Harmison, 2011).

Sport psychology is in many ways a comprehensive scientific field as it provides an arena for studying the interaction of human performance, thoughts and emotions (Singh et al., 2009). However, the sub-field of ski jumping research is small, with only a few research groups focusing on biomechanical, physiological, and aerodynamically related aspects for optimal ski jumping performance (e.g., Virmavirta and Komi, 1993; Komi and Virmavirta, 2000; Ettema et al., 2005; Schwameder, 2008). Thus, there is by the authors knowledge no research on the psychological and emotional aspects of world class ski jumping performance that has been published. The factors investigated in the current study originate from established theories and standardized scales; self-efficacy, flow, positive- and negative affect, and worry. These psychological factors are chosen because they have been consistently associated with successful performances in other sports (Wurtele, 1986; Martens et al., 1990; Jackson and Roberts, 1992; Tenenbaum and Eklund, 2007).

One of the most essential psychological factors influencing performance is self-efficacy (Bandura, 1986; Marsh, 1993; Grant and Greene, 2004). Self-efficacy as a construct, is related to social cognitive theory (SCT) which emphasizes the view of human sense of agency as proactively engaged in their own development, and who actively control their actions (Bandura, 1986). Self-efficacy refers to specific aspects of the self, and what it is capable to do with their abilities and skills. Bandura(1997, p. 3) defined self-efficacy as follows: “*beliefs in one's capabilities to organize and execute the courses of action required to produce given attainments.”* One way in which self-efficacy can influence performance in sport, is through its potential role in regulating emotional states (Bandura, 1997; Feltz et al., 2008). Like physiological arousal, emotional arousal is also of significance for self-efficacy. Furthermore, efficacy beliefs are suggested to impact upon both positive and negative affectivity. More specifically, athletes with high levels of self-efficacy are assumed to have a greater extent of positive emotions, such as happiness, enjoyment, and satisfaction, than are athletes with low levels of self-efficacy that are assumed to have higher levels of negative emotions (e.g., sadness, anxiety, and depression) (Treasure et al., 1996). Schunk (1995) proposed that symptoms that signal anxiety related emotions might be interpreted by the individual (athlete) to believe that he or she lacks the required skills to perform a certain task. This will further influence the athlete's efficacy judgements, and hence, also the performance outcome.

Flow is described as an optimal psychological state of consciousness in which a person has clear goals and is confident in their abilities, is fully concentrated, is getting a sense of control, and become totally absorbed in the activity one is doing (Csikszentmihalyi, 1990). The acquired experiences during this process are positive and joyful. The activity is perceived as inner motivated, autotelic, which means that it is valuable in itself, regardless of the final product or other external benefits the activity may be associated with (Csikszentmihalyi, 1997).

The most crucial prerequisite for experiencing flow is according to Csikszentmihalyi (1997) the positive balance between perceived capacity to act (skill) and perceived opportunity to act (challenge). If your skills do not match the challenges, this leads to uncertainty; and if your skills exceed the challenges, this leads to boredom (Jackson and Csikszentmihayli, 1999). The challenge cannot be too easy nor too difficult yet challenging enough that one knows that to master it, one has to do their very best. For achieving the best possible result, this means that one must maximize both their physical and mental prerequisites, which may be assumed to depend on the attention toward the challenge at hand. The phenomenon of flow is often associated with peak performance in sports, and elite athletes who are achieving outstanding results are more likely to have experienced flow states (Jackson and Roberts, 1992). For example, this was also found in a study of elite golfers during high level performances (Swann et al., 2016).

Further, it may be argued that the abovementioned factors are related to cognitive aspects of emotions and emotional functioning. Emotions are, on the other hand, difficult to define because of high degree of complexity and a substantial range of possible elements. Most theorists agree, however, that an emotion consists of cognitive processes, physiological changes and behavioral aspects (Mann, 1999). Affect is thus often used as a generic term for emotions, moods and emotionally charged assessments (Gray and Watson, 2001). Affect is defined as a person's immediate physiological response to a stimulus and are primarily based on an underlying recollection of arousal which is positive or negative (Snyder and Lopez, 2007). Emotions can be defined as sudden changes of consciousness due to external or internal stimuli, such as anger, pride or shame (Parkinson, 1995). In line with The Cognitive Evaluation Theory (CET), individuals consider first whether an event is relevant to themselves or not. If the event is relevant the individual will continue to assess whether the situation is advantageous, disadvantageous, challenging or threatening to its values, wellbeing or achievement of goals (Lazarus and Folkman, 1984). If the event is threatening or seems challenging, it takes a more comprehensive assessment of the possibilities to change the situation. How an athlete is coping stressors in performance contexts are seen as a process of continuous assessment, reassessment and action. This may involve avoiding, accepting, or minimize them, as well as an attempt to master. The subjective experience of own resources and situational factors are taken into consideration when presented, and this assessment is believed to amplify, confirm or reduce the original experience of the situation (Lazarus and Folkman, 1984). It can therefore be argued that specific emotions will emerge as a result of these subjective assessments, and the perception of coping options will be critical for the athlete's meeting with potential stressors in performance situations.

As mentioned above, our behavior is affected by emotions (Lazarus and Folkman, 1984). Experienced emotions before, during and after competition has been found as a very crucial factor in athletic performance (Hanin, 2010). In order to perform at a high level, it is crucial to handle and control all the intense affects or emotions that are arising in a competitive context (Tenenbaum and Eklund, 2007). For top athletes, e.g., World Cup ski jumpers, the ability to manage and regulate negative emotions, such as anxiety, stress, worry, and expectations, is thus believed to be a very crucial mental skill in competitive situations, and hence, for the performance outcome (Hanin, 2007). Through self-reporting, athletes have identified “normal nervousness” and optimal emotional activation (arousal) to be the best fit associated with high-level performance in other sports, for example, Olympic wrestling (Gould et al., 1992). Inexpedient or negative emotional states, however, has been associated with weaker performances (Tenenbaum and Eklund, 2007). Athletes have also identified key components of mental resilience in performance context (Jones et al., 2002). This consists of the ability to push the limit of physical and emotional pain away, in order to maintain effective performance in stressful situations. Thus, in the context of athletic performance, psychological factors that regulate emotional states can be expected to have both positive and negative effects.

Based on the presented considerations, the specific aims of the current study were to explore the relationship between self-efficacy, flow, affect, worry and performance in World Cup ski jumping, by means of overall ranking and competition results comparison. Social Cognitive Theory (SCT) (Bandura, 1997) and Flow Theory (Csikszentmihalyi, 1990) were used as main conceptual frameworks. In line with this, it was expected to find significant relationships between, and influential functions of, these psychological factors and ski jumping performance.

## Methods

### Participants

The sample (*N* = 40) consisted of male World Cup ski jumpers from 11 different nations, representing some of the elite ski jumpers in the world (see Table 1 for demographics). This follows from their ranking in the overall World cup, which ranged from 2nd to 75th place. Consequently, the sample can be expected to be homogenous in some degree, as World Cup ski jumpers theoretically have relatively comparable technique, fitness (e.g., leg power), and flying abilities (e.g., V-style, body shape etc.) as the basis for participation in the World Cup. All participants provided written informed consent before participating in the study, and the study protocol was approved by the Norwegian Centre for Research Data. The study was conducted in accordance with the declaration of Helsinki.

**Table 1:** Descriptive statistics of the sample (*N*).

**Variable**	***N***	**Min**	**Max**	***M***	***SD***
Age (years)	40	19	36	25	4
Height (cm)	40	168	190	178	5
Weight (kg)	35	55	70	62	4

*Min, Minimum; Max, Maximum; M, Means; and SD, standard Deviations*.

### Instruments

Three of the instruments used in this study were based on previously developed standardized scales [Flow State Scale (FSS; Jackson and Marsh, 1996), Positive- and Negative Affect Schedule (PANAS: Watson et al., 1988), and Penn State Worry Questionnaire (PSWQ; Meyer et al., 1990)]. One was developed for the purpose of this study (Self-Efficacy; based on Bandura, 2006). The three standardized scales used a five-point Likert scale, ranging either from “strongly disagree” (1) to “strongly agree” (5), from “not at all typical of me” to “very typical of me,” or from “very slightly/not at all” to “extremely.” The self-efficacy scale used a seven-point Likert scale, ranging from small degree of certainty (1) to high degree of certainty (7).

#### Self-efficacy

In line with Bandura (2006) “Guide for constructing self-efficacy scales,” an 11-item scale was developed to measure self-efficacy related to specific ski jumping capabilities which were viewed as prominent for performance (see Appendix A). These specific capabilities involve efficacy beliefs concerning the “cycle of ski jumping”—from one jump to the next. These items focused on three different areas representing relevant challenges in performing the sport, and efficacy beliefs toward; equipment, technique and stress management (Bandura, 2006). Examples from the scale would represent phrases like: “*How sure are you that you can perform at your best under pressure (e.g., leading after first round in a World Cup competition)?”* Participants were asked to subjectively consider how sure they were about managing challenges and situations on the previously described seven-point Likert scale.

#### Flow

The 36 – item, Flow State Scale (FSS) developed by Jackson and Marsh (1996), was used to measure optimal experience (flow) among world class ski jumpers. As previously mentioned, flow, and the FSS, consists of nine dimensions with internal consistency estimates of 0.83 for the 36 – item version (Jackson and Marsh, 1996). The participants were asked to respond to the flow items using a five-point Likert type response format as previously described. For example, (Emotion): “*I loved the feeling of that performance and want to capture it again,”* (Focus): “*My attention was focused entirely on what I was doing,”* (Arousal): “*I was not worried about my performance during the event,”* or (Time): “*The way time passed seemed to be different from normal.”* Further, higher scores on the sub scales was reflecting higher levels of flow experiences. The Cronbach's alpha of the instruments is shown in **Table 3**.

#### Positive- and negative affect

The Positive and Negative Affect Schedule (PANAS) consists of two 10-item self-report mood scales and was developed to provide brief measures of positive (PA) and negative (NA) affect. The internal consistencies of the PANAS PA and NA are estimated to be 0.89 for the PA scale, and 0.85 for the NA scale (Crawford and Henry, 2004). This scale was developed by Watson et al. (1988), and in this study, it was used to measure the ski jumper's subjective affective state between World Cup competitions. The participants were asked to rate the extent to which they have experienced each particular emotion with reference to a five-point Likert scale ranging from very slightly or not at all (1), to extremely (2). For example, (PA): “*strong,” “enthusiastic,”* or “*proud,”* and (NA): “*nervous,” “scared,” or “jittery.”* Further, higher scores are reflecting higher levels of either positive or negative affectivity.

#### Worry

The Penn State Worry Questionnaire (PSWQ; Meyer et al., 1990) consists of 16 items. Each item is describing elements of excessive or uncontrollable worry, ranging from “not at all typical of me” (1) to “very typical of me” (5), and thereby yielding a possible range of scores from 16 to 80. In scoring the PSWQ, reversed items need to be checked. Item 1, 3, 8, 10, and 11 are reversed scores. In the original psychometric study using PSWQ on a normal population it was found high internal consistency (α = 0.93) and high test-retest reliability (≥0.74) (Meyer et al., 1990). The PSWQ was used to measure ski jumpers' levels of worry in a competitive context, between World Cup competitions during the season. For example, the PSWQ would represent phrases like: “*When I am under pressure I worry a lot,” “I notice that I have been worrying about things,”* or “*Once I start worrying, I cannot stop.”* The higher the score, the higher levels of worry are reflected. The PSWQ was treated as a one-dimensional scale, as all the questions involved the same underlying factor, worry.

### Procedures

The data collection was conducted during a weekend with three ski flying competitions held in three consecutive days. These competitions were an integral part of the World Cup season, in which consisted of in total 34 competitions. The ski flying competitions takes place between six to ten times within the World Cup and is of considerable importance for the overall World Cup ranking. Indeed, high correlations between ski flying results and overall World Cup ranking was found in the current study (see **Table 3**). It should be noted however, that ski flying and ski jumping might be considered as two different sports. The best normal hill ski jumpers might not be amongst the best in ski flying, due to technical and psychological differences. The main difference between ski flying and ski jumping is the size of the hill. Ski flying hills range between hill sizes from 200 to 240 m, while other ski jumping hills range between hill sizes from 100 to 145 m. In all competitions, two competition rounds are conducted in which the best forty (in ski flying) and the best fifty (in normal ski jumping) ski jumpers qualify for the first round. The qualification round happens the day before actual competition or as a trial round the day of competition. Only the ski jumpers with the thirty highest scores qualify for the final round in a single competition. The results from the individual competitions are measured in positions compared to other ski jumpers based on the total score from each competition (e.g., 1st place, 2nd place, 3rd place, etc.) The overall World Cup ranking is a point system based on the results from all World Cup competitions during a whole season, regardless of the hill size. Only the thirty best ski jumpers are assigned ranking points based on their individual results. For example, the winner from each competition gets 100 points, the second place gets 80 points, the third place gets 60 points, etc. (for more information see: FIS International Competition Rules (ICR) Ski Jumping, 2017).

Seventy one athletes where handed out envelopes that contained questionnaires, information about the research project, and declaration of consent, in which 40 were returned for analysis. The questionnaires where completed once by each athlete, shortly after one out of the three ski flying competitions during the weekend. As not all athletes qualified for the first round in all ski flying competitions, the number of participants with final results varied across the three individual competitions. No athletes experienced any adverse events (e.g., falls/bad landings) during the weekend, and the national team head coaches from the World Cup nations, and the race director of ski jumping in the International Ski Federation (FIS) were informed and approved the current study.

### Data analysis

The data were analyzed using IBM SPSS Statistics (version 24.0, IBM, New York, US). The occurrence of missing values was low (7.5%) and was treated by pairwise exclusion. Three types of data analysis were done. Despite evidence of the factorial validity of the questionnaires related to the constructs of self-efficacy, flow, worry and affect (see e.g., references), the versions of the inventories were firstly subjected to exploratory factor analysis in order to confirm the expected factor structures and estimation of Cronbach's alpha for assessment of internal consistency. Based upon the principal component analysis (PCA) with Varimax rotations, eight indexes were computed from the four questionnaires (see **Table 3**). Secondly, a bivariate correlational analysis using coefficient *r* of Pearson, was conducted on all constructs to demonstrate the relationship between the four psychological factors (self-efficacy, flow, affect and worry) and ski jumping performance, both from the overall World Cup ranking and from the individual events. Lastly a stepwise multiple regression analysis was done to find whether the ski jumping performances (results) could significantly be predicted by the psychological factors, both from the overall World Cup ranking and from the individual events. The significance level was set at the *p* < 0.05.

## Results

Descriptive statistics can be found in Table 2.

**Table 2:** Descriptive statistics of study variables.

**Variable**	***N***	**Cronbach's α**	***M***	***SD***
Ski flying comp 1 (Pos.)	40		29.5	16.2
Ski flying comp 2 (Pos.)	23		18.4	11.1
Ski flying comp 3 (Pos.)	24		18.5	11.6
Overall WC (Pos.)	40		38.0	25.9
SE-Ski jumping (11 items)	40	0.91	56.8	9.8
Flow-Emotion (14 items)	40	0.95	48.4	10.5
Flow-Focus (12 items)	39	0.93	46.2	8.0
Flow-Arousal (5 items)	40	0.78	16.0	3.8
Flow-Time (4 items)	39	0.82	10.9	3.1
Positive affect: PA (10 items)	38	0.82	34.3	6.5
Negative affect: NA (9 items)	39	0.84	20.5	6.4
PSWQ (Worry) (16 items)	38	0.89	43.3	10.5

*M, Means; SD, Standard deviations; Pos., Position in competition/world cup overall (WC)*.

### Factor analysis

Initial analysis indicated that all four questionnaires demonstrated acceptable values of sampling adequacy to conduct factor analysis: significant Bartlett's tests of sphericity (*p* < 0.05), and with Kaiser-Meyer-Olkin (KMO) measures greater- or slightly less than 0.6 (Self-Efficacy = 0.78, Flow State Scale = 0.56, Positive- and Negative Affect Schedule: 0.72, Penn State Worry Questionnaire = 0.71).

#### Self-efficacy

Exploratory factor analysis using the varimax rotation returned a one-factor solution with an eigenvalue of 5.71, accounting for 51.9% of the total variance, and with a high Cronbach's alpha (0.91). The participants' scores yielded a possible range from 11 to 77, with higher scores reflecting higher levels of self-efficacy. There were no missing values on this scale.

#### Flow

The PCA, using the varimax rotation, returned a four-factor solution of flow with eigenvalues: >1 (Flow-Focus: 13.96, Flow-Emotion: 3.17, Flow-Arousal: 2.74, Flow-Time: 2.59), that explained 64.2% of the total variance. All sub scales had different numbers of items, depending on the factor loadings, and one item was discarded due to nearly equal loading to all other sub scales (item 11 in the FSS). Thus, the total score in the FSS yielded a possible range from 35 to 175, with higher scores reflecting increasing levels of flow experience. Two missing values was found and replaced with mean.

#### Positive and negative affect

The PCA from the Positive- and Negative Affect Schedule (PANAS) returned a two-factor solution, as expected, with positive- and negative affect, respectively (PA, NA). Eigenvalue PA = 3.17, NA = 5.58, explaining 46.1% of the total variance. Due to equal loadings from the PCA, and/or misinterpretation among participants, one item was discarded (item 19 “Active” in the PANAS) leaving the NA subscale with nine items, and the PA subscale with ten items. This generates a possible range of scores from 9 (NA) or 10 (PA) to 45 (PA) or 50 (NA) respectively, with higher scores reflecting higher levels of either positive- or negative affect. One participant did not respond to any of the items in the PANAS and hence, discarded. The whole scale was used (1–5).

#### Worry

The PCA from the Penn State Worry Questionnaire (PSWQ) returned a one-factor solution with an eigenvalue of 6.47, explaining 40.4% of the total variance and high Cronbach's alpha (.89). The participants' scores yielded a possible range from 16 to 80 (controlled for reversed items), with higher scores reflecting higher levels of worry. One participant did not respond to any of the items in the PSWQ.

### Ski jumping performance

#### Correlational analysis

The coefficients from the correlational analysis (see Table 3) indicate significant associations between overall World Cup (WC) ranking and self-efficacy (SE-Ski jumping), and between overall WC ranking and Flow (Flow-Focus). There were no significant correlations between overall WC ranking and other factors (*p* > 0.05).

**Table 3:** Correlations (Pearson) between the ski jumping results and scores from the psychological factors (WC ski jumpers, valid *n* = 39).

**Indexes**	**1**	**2**	**3**	**4**	**5**	**6**	**7**	**8**	**9**	**10**	**11**	**12**
1. Overall WC ranking	1	0.693^**^	0.880^**^	0.609^**^	−0.374^*^	−0.185	−0.337^*^	−0.115	0.031	−0.293	0.279	0.297
2. Results-Ski flying Comp 1		1	0.802^**^	0.773^**^	−0.357^*^	−0.165	−0.293	−0.361^*^	0.077	−0.141	0.335^*^	0.289
3. Results-Ski flying Comp 2			1	0.770^**^	−0.362^*^	−0.201	−0.444^*^	−0.271	−0.241	−0.477^*^	0.044	0.603^**^
4. Results-Ski flying Comp 3				1	-0.214	0.001	−0.227	−0.244	−0.026	−0.339	−0.027	0.522^*^
5. SE_Index (self-efficacy: SE-ski jumping)					1	0.325^*^	0.654^**^	0.253	−0.034	0.297	−0.585^**^	−0.265
6. FSS_Emotion (Flow-Emotion)						1	0.698^**^	0.381^*^	0.243	0.588^**^	−0.203	−0.049
7. FSS_Focus (Flow-Focus)							1	0.479^**^	0.249	0.599^**^	−0.459^**^	−0.312
8. FSS_Arousal (Flow-Arousal)								1	0.205	0.188	−0.271	−0.390^*^
9. FSS_Time (Flow-Time)									1	0.396^*^	0.043	−0.050
10. PANAS_PA (Positive affect: PA)										1	−0.289	−0.246
11.PANAS_NA (Negative affect: NA)											1	0.445^**^
12. PSWQ (Worry)												1

** *Correlation is significant at the 0.01 level (2-tailed)*.

* *Bold values signify significant correlations*.

There were also significant associations between ski flying results and self-efficacy (SE-Ski jumping), Flow (Flow-Arousal), and negative affect (NA) in competition 1. In competition 2 there were significant associations between ski flying results and self-efficacy (SE-Ski jumping), Flow (Flow-Focus), positive affect (PA) and worry (PSWQ). In competition 3 the significant associations were only found between the ski flying results and worry (PSWQ). There were no significant correlations between ski flying results and other factors (*p* > 0.05) (see Table 3), or between the age of the athletes and the psychological factors (*p* > 0.05).

The analysis shows however, that there are negative correlations between results and some of the variables. This, however, is misleading because it really shows that higher scores on the scales means that the respondents are higher up on the ranking list, and vice versa from the positive correlation. Interestingly, there were found correlations between several subscales as well (see Table 3).

#### Regression analysis

A stepwise multiple regression analysis indicated a multiple correlation coefficient of 0.37, indicating that approximately 14% of the variance of the ranking in the overall World Cup could be accounted for by the psychological factor self-efficacy (SE-Ski jumping) [*F*_(1, 37)_ = 6.01, *p* = 0.019, $R_{\text{Adjusted}}^{2}$ = 0.12]. It was found that self-efficacy significantly influenced overall World Cup ranking (β = −0.37, *p* = 0.019) in this study. All other variables' scores did not enter significantly into the equation at step 2 of the analysis [*p* > 0.05: Flow-Focus, Flow-Emotion, Flow-Arousal, Flow-Time, Positive affect (PA), Negative affect (NA), and PSWQ (Worry)].

Similarly, a stepwise multiple regression analysis was conducted to test whether the psychological factors significantly could predict the participants' individual results from the individual ski flying competitions during the weekend. In competition 1, the multiple correlation coefficient was 0.36, indicating that approximately 13% of the variance of the individual ski flying competition results could be accounted for by the Flow State Scale index, the Flow-Arousal [*F*_(1, 37)_ = 5.54, *p* = 0.024, $R_{\text{Adjusted}}^{2}$ = 0.11]. It was found that the Flow-Arousal index significantly influenced the results from the first ski flying competition (β = −0.36, *p* = 0.024) in this study. All other variables' scores did not enter into the equation at step 2 of the analysis (*p* > 0.05: SE-Ski jumping, Flow-Emotion, Flow-Focus, Flow-Time, Positive affect (PA), Negative affect (NA), and PSWQ (Worry).

In competition 2 and 3 the multiple correlation coefficient was 0.60 and 0.52, respectively, indicating that approximately 36 and 27% of the variance in ski flying results could be accounted for by levels of worry (PSWQ) [*F*_(1, 20)_ = 11.43, *p* = 0.003, $R_{\text{Adjusted}}^{2}$ = 0.33], [*F*_(1, 21)_ = 7.88, *p* = 0.011, $R_{\text{Adjusted}}^{2}$ = 0.24]. It was found that the PSWQ index significantly influenced the ski flying results in day 2 and 3 of this ski flying weekend (β = 0.60, *p* = 0.003), (β = 0.52, *p* = 0.011) respectively, in this study.

## Discussion

The primary aim of this study was to explore the association between self-efficacy, flow, positive- and negative affect and worry and performance in elite ski jumping. The findings suggest that there are moderate correlations between overall world cup ranking, self-efficacy and flow (Flow-Arousal). Similarly, self-efficacy and flow (Flow-Focus) appeared as significant predictors of elite ski jumping performance on the first day of competition, whilst worry, appeared as a significant predictor of performance on the second and third competition. Negative affect was moderately related to ski flying performance on the first day of competition.

### Overall world cup ranking

Regression analysis indicated that the responses from the self-efficacy questionnaire containing ski jumping specific items explained 14% of the variance in the overall World Cup ranking. This suggests that this psychological factor in which captures ski jumpers' beliefs in their own capacity to master challenges, may be of significant importance in regard of elite ski jumping performance throughout a whole season.

This result converges with both theory and empirical findings of self-efficacy as one of the most influential factors for athletic performance (Bandura, 1986; Gould et al., 1999; Moritz et al., 2000; Feltz et al., 2008). Those high in self-efficacy tend to try harder, endure longer, choose more demanding challenges, experience the efforts more positively and feel less anxiety, compared to those low in self-efficacy (Mouloud et al., 2015). This suggests however, that in order to perform on a high level throughout a whole World Cup season, high self-efficacy beliefs seem therefore to have a reasonable amount of importance. This may partially be explained by one of Bandura (1997) four sources to self-efficacy. More precisely, the ski jumpers' self-efficacy during the whole season may converge with *past performance accomplishments*. This means that any performances prior to this data collection were constructing future self-efficacy for the next competition, and it seems that it lasted more or less throughout the rest of the season. Thus, past performance accomplishments (or lack of) may be assumed to have an effect on future self-efficacy and future athletic accomplishments, and vice versa.

No other psychological factor significantly influenced performance outcome in the overall World Cup ranking in this study. A potential explanation for the lack of significant influence, may be due to the assumed reciprocal relationship between self-efficacy, flow, affect, worry and their influence on ski jumping performance. Previous research has argued that high self-efficacy, among other dispositional factors, is typically related with pleasant emotions (PA) subjectively perceived as performance enhancing. In contrast, low self-efficacy is normally seen as an antecedent of unpleasant emotions (NA), anxiety and worry, subjectively perceived as inhibitory for the performance outcome (Jones et al., 2009). It may therefore be argued that self-efficacy can be explained as the sum of all other emotionally interfering antecedents, and thereof influencing the performance outcome in the overall World Cup.

### Single ski flying competitions

During three consecutive days of ski flying events, self-efficacy and flow was significantly associated with performance on the first day of competition. Specifically, the part of flow termed Flow-Arousal, explained 13% of the variance in this competition. This may thus indicate that ski flying, which is the most extreme kind of ski jumping, set higher demands to the ski jumpers' sense of control, automaticity and appropriate arousal levels in order to perform on a world class level, compared to other emotionally related factors.

This converges with what was previously mentioned, as flow being a very functional state of consciousness, and as the underlying psychological process for peak performance in sports (Jackson and Roberts, 1992). Similarly, the study conducted by Swann et al. (2016) indicated that elite golfers experienced a gradual build-up of confidence during excellent performances, which corresponded to descriptions of flow. Thus, this notion of flow, focus, automaticity, or “let it happen,” seems to be present in high performance contexts regardless of sport.

However, Flow-Arousal, together with self-efficacy, seemingly had a decreased influence on the performance outcome during the following days of ski flying, whilst worry had an extensive, increased influence. This decrease in Flow-Arousal and self-efficacy might be explained by *past performance accomplishments* (SE), together with physical, emotional and mental strain, as this may allow for a substantial amount of negative affectivity (NA) and thoughts of worry to flourish. Interestingly, regression analysis indicated that the responses from the PSWQ questionnaire containing worry related items, explained 36 and 27% of the variance in ski flying results in competition two and three, respectively. This further suggests that ski jumpers' level of worry may have a substantial impact on ski flying performance as the competitions reaches over numerous days. Ski flying happens relatively rare compared to “normal” ski jumping during a season, and ski jumpers are only allowed to execute a maximum of four jumps a day in ski flying, due to safety reasons. It may thus be reasonable to assume that the athletes' excitement, nervousness, expectations and mental pre-ski flying preparations (e.g., *verbal persuasion*) are increased until the first flights have been executed.

Worry is found to affect attention control, or focus, and thereof inhibit determination control on given tasks (Eysenck et al., 2007). Further, worry has previously been associated with increased cognitive activity. The study of highly skilled golfers with the use of EEG, showed that high performers were thinking less (reduced cognitive activity) than those performing poorly (Crews and Landers, 1993), and this also shares some similarities to previously mentioned descriptions of flow (Csikszentmihalyi, 1990). Based on the data from the current study, the results showed an increase in levels of worry after the first competition. A possible explanation for why worry did not appear as a significant influence on ski flying performance on the first day of competition might be the ski jumpers' capacity to persuade themselves. More specifically, by the use of *verbal persuasion* (Bandura, 1997) as a means to be able to cope with challenging thoughts or tasks (Feltz et al., 2008). All ski jumpers, regardless of level, might be assumed to worry about something, at some point, during the season. It might be argued that when you are competing in the World Cup circuit, and in particular ski flying competitions, you cannot allow yourself to be worried, even if you are. As part of a verbal persuasion strategy, the ski jumpers might be inclined to use positive self- and task-related statements when they are in competitive contexts—even when responding to questionnaires—regardless of performance outcome.

In the following competitions, it may thus be reasonable to assume that the physical, emotional and mental strain these athletes experience in this kind of context, are influencing their cognitive activity to varying degrees, making their own perception of possible coping options critical in performance situations (Lazarus and Folkman, 1984). Furthermore, the athletes who were scoring high on worry in the second and third competition, may have interpreted the situation too challenging, disadvantageous or threatening to their wellbeing or achievement of goals, in accordance with the CET, to be able to properly use a functional verbal persuasion strategy. However, a necessary next step in order to examine these matters further should include an investigation of the best performing ski jumpers' score on worry, and the other psychological factors, during consecutive days of World Cup competitions on the normal, large and flying hills.

### General discussion

The results from this study provide further evidence for the significant impact that flow, self-efficacy, and worry could have on athletic performance (Wurtele, 1986; Martens et al., 1990; Jackson and Roberts, 1992; Tenenbaum and Eklund, 2007). Ski jumpers who perform well can therefore be assumed to hold a greater degree of self-efficacy and flow experiences in a performance context, positively reinforced by appropriate affective states and lower degree of cognitive anxiety, compared to those who perform poorly. Those who are performing poorly may be assumed to experience lower levels of self-efficacy and flow, and to a greater extent be influenced by negative affective states and cognitive anxiety, such as worry. It may thus be reasonable to assume that these psychological factors more or less could be mutually interacting with each other for optimal ski jumping performance. It needs to be addressed however, that the ski jumpers' performance outcome in this study could be associated by an extensive number of other influential factors and that the overall explained variance is moderate.

### Methodological reflections

The results from the factor analyses showed some discrepancy between expected and actual factor loadings. The self-efficacy scale was expected to reveal three dimensions; Equipment, Technique and Stress. However, the eigenvalue and the factor loadings from this analysis made it obvious that it had to be treated as a one-dimension scale (SE-Ski jumping). Additionally, the standardized FSS was expected to reveal the nine sub dimensions of flow, whereas the factorial analysis extracted only four dimensions. This however, made it a bit challenging in the sense of “renaming” factors based on their eigenvalue, and factor loadings. The PANAS and the PSWQ revealed factors as expected.

In the current study we applied overall World Cup ranking, a point system based on individual World Cup results, and the results from three separate ski flying competitions as measures of performance. The first measure represents the overall ranking from 34 separate competitions across the world and consist of primarily two hill sizes during the season, normal large hill ski jumping and ski flying. The reason for applying ski flying and not normal ski jumping in the current study was to promote the indications of which athlete who holds more appropriate coping strategies and a constructive mindset in more psychologically demanding situations, compared to those athletes who do not.

Although there are high inter-correlations between World Cup ranking and ski flying results, an important inquiry for further research would be to address the data collection to normal ski jumping as well, as the athletes' attitude might differ in that context. The data collection should also take place over several periods during the season to avoid random responses and to get insight from different contexts. A qualitative approach could also be recommendable in combination with quantitative data in order to get an even more complementing understanding of the psychological impact on ski jumping performance.

The self-efficacy scale used in the current study was primarily designed to assess the possible psychologically-influencing factors in all the phases of a ski jump. However, the scale did not include all aspects of potential influencing factors, such as the effects of changing environment or different circumstances (wind, snow, fog etc.), previous injuries of the athlete, or the landing phase, among other. These factors could possibly have influenced the athletes' emotional state, either positively or negatively, depending on how one interprets the situation.

As for environmental changes, the FIS proposed a wind compensation score that is applied during official competitions, as a means to make the competitions fairer. If a ski jumper has really good wind conditions he/she will get minus points, and vice versa if the wind conditions are bad (FIS International Competition Rules (ICR) Ski Jumping, 2017). This compensation, as with snow, fog etc., will more or less be equalized in the course of a whole season, even if it can constitute a major impact on performance results in some individual competitions. Thus, further research should emphasize also these factors to enhance the research design.

## Conclusion

The current study explored the association between self-efficacy, flow, affect, worry and various indices of elite ski jumping performance. The findings indicated that overall World Cup ranking after a complete competitive season was moderately related to self-efficacy and flow. Across a weekend of three competitions, the pattern of results suggested that self-efficacy and flow was associated with performance in the first competition, while performance in the second and third competitions was only related to worry. Negative affect did not appear as a significant predictor of any indicator of elite ski jumping performance. Overall, the results of the current study suggest a significant role of the ability to regulate anxiety levels, arousal and adversity in achieving the highest level of elite ski jumping performance. In order to establish a thorough understanding of the putative role of emotional regulation in elite ski jumping, further work should construct and validate conceptual scales more specifically designed to assess emotional functioning in the specific context. Furthermore, larger samples that includes athletes from different levels of performance would allow for further testing of theoretical models with structural equation modeling. Given the significant impact of the coach in elite sport, the putative association between coach behavior/attitudes and the athletes' emotional states also needs to be addressed. This endeavor could possibly lead to psychologically-based performance enhancing strategies in elite ski jumping performance.

## Author contributions

VS, HL, and HS involved in planning, data analysis, and writing of the paper.

### Conflict of interest statement

The authors declare that the research was conducted in the absence of any commercial or financial relationships that could be construed as a potential conflict of interest.
