# Supplementary material for: Self-Efficacy, Flow, Affect, Worry and Performance in Elite World Cup Ski Jumping
Source: Front Psychol. 2018 Jul 17;9:1215. doi: 10.3389/fpsyg.2018.01215 (PMC6057433; doi:10.3389/fpsyg.2018.01215)
Supplement: Supplementary file 1 [file Presentation_1.pdf]

Appendix A

Self-efficacy and ski jumping performance

Introduction: The role of an athlete is complex and holds many different tasks and challenges. The following questions are designed to identify to which degree you, as a world cup ski jumper, strongly believe to master the many challenges you face.
Each question involves how sure you are in performing certain tasks and certain situations during ski jumping.

Instructions: Complete the following scale either before or after a competitive situation that you feel is highly important to you.

Read each question thoroughly and consider how sure you are in managing the tasks in the conditions described and circle the appropriate number to indicate how you feel right now, at this moment. Small degree of certainty = 1, and high degree of certainty = 7

There are no right or wrong answers. Do not spend too much time on any one of the questions.

Small degree of certainty			Moderately degree of certainty			High degree of certainty
1	2	3	4	5	6	7

Equipment

How sure are you...:

1. …that today, your equipment is good enough to reach the highest level of performance in world cup competitions? 1 2 3 4 5 6 7
2. …that your suit is optimal, so you are able to compete with the best in the world? 1 2 3 4 5 6 7
3. …that your equipment gives you confidence in performing at the highest level in world cup? 1 2 3 4 5 6 7

Technique

How sure are you…:

4. …that your in-run position is good enough to reach the highest level of performance in world cup competitions? 1 2 3 4 5 6 7
5. …that your take-off technique is stable, and on a very high level in the world cup? 1 2 3 4 5 6 7

6.	…that you are capable to fly effectively no matter what the outcome from the table is?	1	2	3	4	5	6	7
7.	…that your knowledge about ski jumping is good enough for high level performance (world class)?	1	2	3	4	5	6	7

Stress

How sure are you…:

8.	…that you feel calm and confident under pressure so you can perform on the highest level in world cup?	1	2	3	4	5	6	7
9.	…that you can perform at your best under pressure (e.g. leading after first round in a world cup competition)?	1	2	3	4	5	6	7
10.	…that you can use stressful situations to perform better?	1	2	3	4	5	6	7
11.	…that today, you are capable to be among the best ski jumpers in the world?	1	2	3	4	5	6	7

Appendix B

FLOW STATE SCALE (FSS)

Flow state scale

Please answer the following questions in relation to your experience in the event you have just completed. These questions relate to the thoughts and feelings you may have experienced during the event. There are no right or wrong answers. Think about how you felt during the event and answer the questions using the rating scale below. Circle the number that best matches your experience from the options to the right of each question.

Rating scale:

Strongly disagree	Disagree	Neither agree nor disagree	Agree	Strongly agree
1	2	3	4	5

	Strongly disagree				Strongly agree
1. I was challenged, but I believed my skills would allow me to meet the challenge.	1	2	3	4	5
2. I made the correct movements without thinking about trying to do so.	1	2	3	4	5
3. I knew clearly what I wanted to do.	1	2	3	4	5
4. It was really clear to me that I was doing well.	1	2	3	4	5
5. My attention was focused entirely on what I was doing.	1	2	3	4	5
6. I felt in total control of what I was doing.	1	2	3	4	5
7. I was not concerned with what others may have been thinking of me.	1	2	3	4	5
8. Time seemed to alter (either slowed down or speeded up).	1	2	3	4	5
9. I really enjoyed the experience.	1	2	3	4	5
10. My abilities matched the high challenge of the situation.	1	2	3	4	5
11. Things just seemed to be happening automatically.	1	2	3	4	5
12. I had a strong sense of what I wanted to do.	1	2	3	4	5
13. I was aware of how well I was performing.	1	2	3	4	5
14. It was no effort to keep my mind on what was happening.	1	2	3	4	5
15. I felt like I could control what I was doing.	1	2	3	4	5
16. I was not worried about my performance during the event.	1	2	3	4	5
17. The way time passed seemed to be different	1	2	3	4	5

from normal.					
18. I loved the feeling of that performance and want to capture it again.	1	2	3	4	5
19. I felt I was competent enough to meet the high demands of the situation.	1	2	3	4	5
20. I performed automatically.	1	2	3	4	5
21. I knew what I wanted to achieve.	1	2	3	4	5
22. I had a good idea while I was performing about how well I was doing.	1	2	3	4	5
23. I had total concentration.	1	2	3	4	5
24. I had a feeling of total control.	1	2	3	4	5
25. I was not concerned with how I was presenting myself.	1	2	3	4	5
26. It felt like time stopped while I was performing.	1	2	3	4	5
27. The experience left me feeling great.	1	2	3	4	5
28. The challenge and my skills were at an equally high level.	1	2	3	4	5
29. I did things spontaneously and automatically without having to think.	1	2	3	4	5
30. My goals were clearly defined.	1	2	3	4	5
31. I could tell by the way I was performing how well I was doing.	1	2	3	4	5
32. I was completely focused on the task at hand.	1	2	3	4	5
33. I felt in total control of my body.	1	2	3	4	5
34. I was not worried about what others may have been thinking of me.	1	2	3	4	5
35. At times, it almost seemed like things were happening in slow motion.	1	2	3	4	5
36. I found the experience extremely rewarding.	1	2	3	4	5

Appendix C

PANAS

This scale consists of a number of words that describe different feelings and emotions. Read each item and then mark the appropriate answer in the space next to the word. Indicate to what extent you have felt like this before, after or during a recent world cup competition. Use the following scale to record your answers.

Very slightly or not at all	a little	moderately	quite a bit	extremely
1	2	3	4	5

Interested	_____	Irritable	_____
Distressed	_____	Alert	_____
Excited	_____	Ashamed	_____
Upset	_____	Inspired	_____
Strong	_____	Nervous	_____
Guilty	_____	Determined	_____
Scared	_____	Attentive	_____
Hostile	_____	Jittery (anxious)	_____
Enthusiastic	_____	Active	_____
Proud	_____	Afraid	_____

Appendix D

The original version of the Penn State Worry Questionnaire

Instruction: Enter the number that best describes how typical or characteristic each item is of you, putting the number next to each item.

1	2	3	4	5
**Not at all typical**		**Somewhat typical**		**Very typical**

____ 1. If I don't have enough time to do everything, I don't worry about it.
____ 2. My worries overwhelm me.
____ 3. I don't tend to worry about things.
____ 4. Many situations make me worry.
____ 5. I know I shouldn't worry about things, but I just can't help it.
____ 6. When I'm under pressure, I worry a lot.
____ 7. I am always worrying about something.
____ 8. I find it easy to dismiss worrisome thoughts.
____ 9. As soon as I finish one task, I start to worry about everything else I have to do.
____ 10. I never worry about anything.
____ 11. When there is nothing more I can do about a concern, I don't worry about it anymore.
____ 12. I've been a worrier all my life.
____ 13. I notice that I have been worrying about things.
____ 14. When I first start worrying, I can't stop.
____ 15. I worry all the time.
____ 16. I worry about projects until they are all done.
